# Supplementary material for: SRST2: Rapid genomic surveillance for public health and hospital microbiology labs
Source: Genome Med. 2014 Nov 27;6(11):90. doi: 10.1186/s13073-014-0090-6 (PMC4237778; doi:10.1186/s13073-014-0090-6)

Accuracy of allele calling using SRST2 vs assembly and BLAST

MLST analysis of public data from 5 species (N=543 genomes, 3801 loci, details Supplementary Table 1). Tests were grouped by read depth and accuracy rates (left y-axis, correct allele calls as a proportion of tests), calculated at each depth (x-axis, red slashes indicate scale change). Grey bars, number of tests at each depth (right y-axis); Lines, accuracy of allele calling. (**A**) Call rate (total allele calls / 3801). (**B**) True positive rate (correct allele calls / total allele calls).

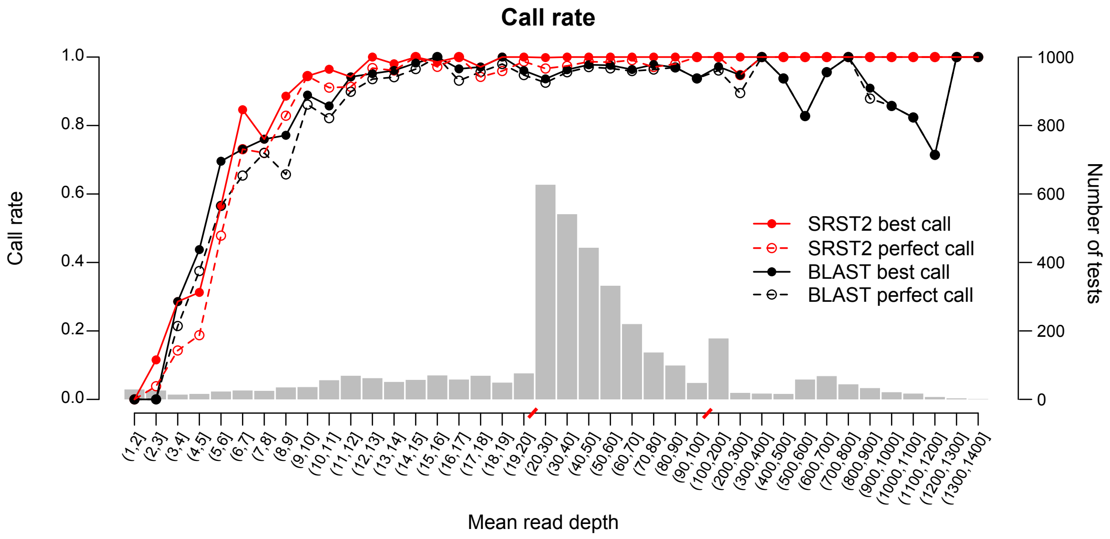

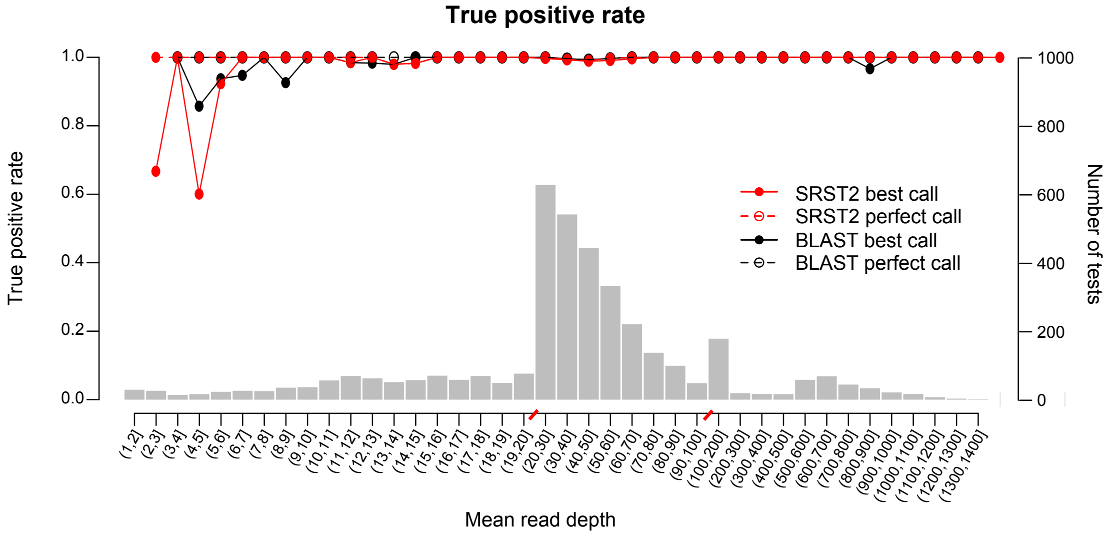

Supplement: Additional file 2 — Separate plots for call rates and true positive rates for the six public data sets used for MLST allele typing validation (these two measures were combined to give the overall accuracy plot in Figure 3 ). [file 13073_2014_90_MOESM2_ESM.pdf]
